# Investigating the effect of arachidonate supplementation on the phosphoinositide content of MCF10a breast epithelial cells

**DOI:** 10.1016/j.jbior.2015.11.002

**Published:** 2016-09

**Authors:** Karen E. Anderson, Veronique Juvin, Jonathan Clark, Len R. Stephens, Phillip T. Hawkins

**Affiliations:** a Inositide Laboratory and Babraham Institute, Babraham Research Campus, Babraham, Cambridge, CB223AT, UK; b Biological Chemistry Laboratory, Babraham Institute, Babraham Research Campus, Babraham, Cambridge CB223AT, UK

**Keywords:** Arachidonate, Phosphoinositides, MCF10a cells, PIP_3_

## Abstract

Phosphoinositides in primary mammalian tissue are highly enriched in a stearoyl/arachidonyl (C38:4) diacylgycerol backbone. However, mammalian cells grown in culture typically contain more diverse molecular species of phosphoinositides, characterised by a reduction in arachidonyl content in the sn-2 position. We have analysed the phosphoinositide species in MCF10a cells grown in culture by mass spectrometry. Under either serum or serum starved conditions the most abundant species of PI, PIP, PIP_2_ and PIP_3_ had masses which corresponded to C36:2, C38:4, C38:3, C38:2 and C36:1 diacylglycerol backbones and the relative proportions of each molecular species were broadly similar between each phosphoinositide class (approx. 50%, 25%, 10%, 10% and 10% respectively, for the species listed above). Supplementing the culture medium with BSA-loaded arachidonic acid promoted a rapid increase in the proportion of the C38:4 species in all phosphoinositide classes (from approx. 25%–60% of total species within 24 h), but the total amount of all combined species for each class remained remarkably constant. Stimulation of cells, cultured in either normal or arachidonate-enriched conditions, with 2 ng/ml EGF for 90 s caused substantial activation of Class I PI3K and accumulation of PIP_3_. Despite the increased proportion of C38:4 PIP_3_ under the arachidonate-supplemented conditions, the total amount of all combined PIP_3_ species accumulating in response to EGF was the same, with or without arachidonate supplementation; there were however small but significant preferences for the conversion of some PIP_2_ species to PIP_3_, with the polyunsaturated C38:4 and C38:3 species being more favoured over other species. These results suggest the enzymes which interconvert phosphoinositides are able to act on several different molecular species and homoeostatic mechanisms are in place to deliver similar phosphoinositide pool sizes under quite different conditions of arachidonate availability. They also suggest enzymes regulating PIP_3_ levels downstream of growth factor stimulation (i.e. PI3Ks and PIP_3_-phosphatases) show some acyl selectivity and further work should be directed at assessing whether different acyl species of PIP_3_ exhibit differing signalling potential.

## 1 Introduction

**Fig. 1:**
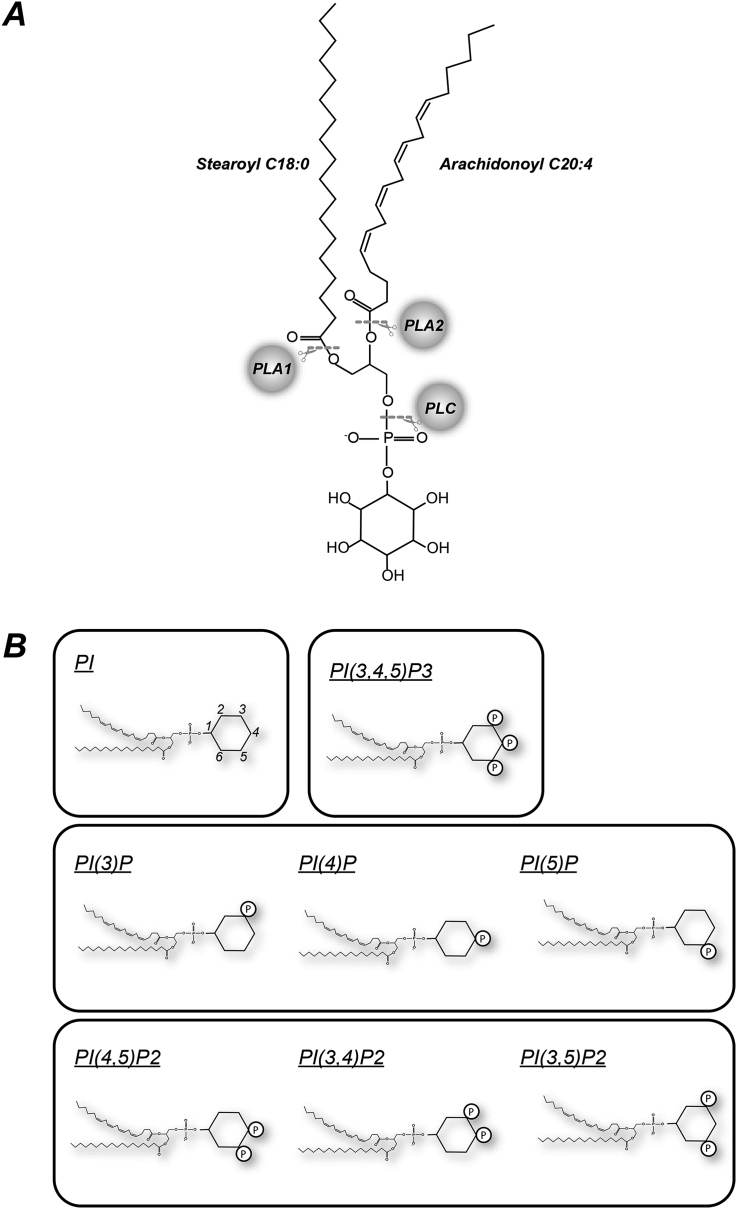
Phosphoinositide structures. A: The structure of stearoyl (C18:0), arachidonyl (C20:4) phosphatidylinositol, together with the bonds cleaved by phospholipases A1, A2 and C. B: Cartoon structures for the phosphoinositides found in higher eukaryotes.

**Fig. 2:**
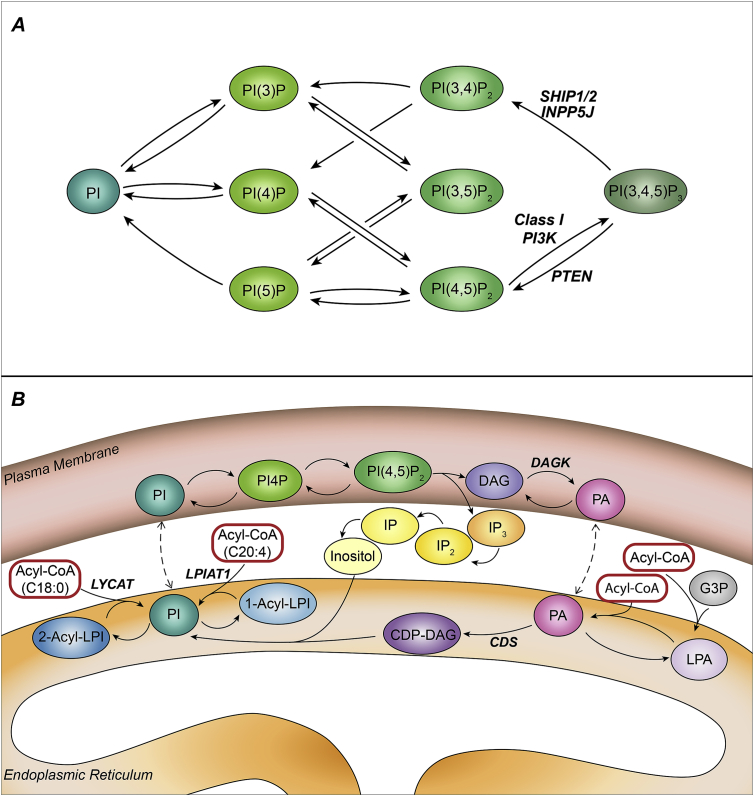
Pathways of phosphoinositide metabolism. A: Pathways for inter-conversion of phosphoinositides by lipid kinases and phosphatases. Enzyme activities mentioned in the text are highlighted. B: A schematic illustration of the major pathways for *de novo* synthesis of phosphoinositides in higher eukaryotes. Enzyme activities highlighted indicate points at which enrichment for C38:4 species may occur.

Phosphoinositides are a family of inositol-containing phospholipids that comprise eight different headgroups in higher eukaryotes; PI, PI(3)P, PI(4)P, PI(5)P, PI(4,5)P_2_, PI(3,4)P_2_, PI(3,5)P_2_ and PI(3,4,5)P_3_ (PIP_3_) (see Fig. 1A) (Balla, 2013). They are interconverted by kinases and phosphatases that add or remove mono-esterified phosphate groups on the inositol ring (Fig. 2A) (Balla, 2013). They can also be metabolised by phospholipases A, C or D that remove acyl chains or inositol headgroups (Fig. 1B). PI is synthesised *de novo* in a specialised compartment of the endoplasmic reticulum (Fig. 2B) and shuttled between membranes by exchange proteins, but enzymes responsible for phosphorylation and dephosphorylation of PI, PIPs and PIP_2_s exhibit widely different activities at different sub-cellular locations, leading to widely differing distributions for the polyphosphorylated phosphoinositides themselves (Balla, 2013). The different phosphoinositides have been ascribed multiple regulatory roles on the membranes in which they are found, and a common theme is that they act as regulatable scaffolds that dictate the location and activity of effectors through the specific binding of their differentially phosphorylated inositol head groups to conserved protein domains (Hammond and Balla, 2015, Lemmon, 2008). One of the best studied roles for these lipids is the stimulated conversion of PI(4,5)P_2_ to PIP_3_ at the plasma membrane by growth factor receptor activation of Class I PI3Ks (Fig. 2A) (Hawkins and Stephens, 2015, Vanhaesebroeck et al., 2010). PIP_3_ acts as a signal by co-ordinating the activity of multiple effector proteins, which in turn regulate complex cellular responses such as cell growth and movement. The best studied of these PIP_3_ effectors are PH-domain containing protein kinases (e.g. AKT, PDK1, BTK) and GEFs or GAPS for small GTPases (e.g. P-REX, GRP-1, ARAP3) (Hawkins and Stephens, 2015, Vanhaesebroeck et al., 2010). Mutations driving increased activity of Class I PI3K and/or decreased removal of PIP_3_ are amongst the most prevalent in a range of human cancers, including EGF-receptor dependent transformation of breast epithelial cells (Wilks, 2015, Yap et al., 2015).

Individual classes of glycerophospholipids are defined by the nature of the head group present, but they usually comprise a surprisingly diverse range of molecular species that are differentiated by the nature of the hydrocarbon chains linked via acyl or ether linkages to their glycerol backbone. The precise range of chains present is dictated both by the cell type and the head group, but typically comprises more than 5–30 different species for one head group. In most cases the biological function of this hydrocarbon diversity is unknown and is usually assumed to reside in the aggregate biophysical properties that the sum total of these species convey to the membranes in which they reside, such as fluidity or bilayer dimension. The phosphoinositides are unusual amongst glycerophospholipids in that they have been found to be much more molecularly homogenous than other analogous lipid classes; thus phosphoinositides in mammals are typically >80% stearoyl (C18:0)/arachidonyl (C20:4) phosphatidylinositols (see Fig. 1) (Anderson et al., 2013, Hicks et al., 2006, Lee et al., 2012). This suggests some evolutionary conserved function might be ascribed to this particular species that may relate to their signalling roles e.g. movement within the plane of a bilayer, or the manner of head group presentation to effectors.

Although phosphoinositides found in primary mammalian tissue are largely the C38:4 species, several studies have suggested cells grown in culture are deprived of arachidonate and contain a more molecularly diverse repertoire of acyl chains (Clark et al., 2011, Rouzer et al., 2006). We sought to investigate the effect of enriching the culture medium of MCF10a breast epithelial cells with arachidonate on the acyl chain composition of phosphoinositides and the quantity of PIP_3_ accumulating upon activation of PI3K signalling by EGF.

## 2 Materials and methods

### 2.1 Materials

Trimethylsilyl-diazomethane (as a 2 M solution in hexanes), arachidonate, fatty acid free (FAF)- and endotoxin free- BSA were from Sigma–Aldrich. 1-heptadcanoyl-2-hexadecanoyl-sn-glycero-3-(phosphoinositol-3,4,5-trisphosphate) (C17:0/C16:0-PI(3,4,5)P_3_, as a hepta-sodium salt) used as an internal standard for mass spectrometry (ISD), was made at the Babraham Institute as previously described (Clark et al., 2011). All cell culture reagents were from Invitrogen, while all other reagents, unless specified, were from Sigma.

### 2.2 Arachidonic acid preparation and supplementation

A solution of arachidonic acid (50 mM) was prepared in 10 mM KOH, and degassed by freeze/thawing under vacuum three times. This solution was then diluted 67.26 fold in Dulbecco's PBS supplemented with 0.862 mM fatty acid free-, essentially endotoxin free- BSA, pH7.4 which had been similarly degassed, to give a stock concentration of 740 μM (50× final). The final stock was aliquoted under argon and stored at −80 °C. Immediately prior to use, aliquots were thawed and added to horse serum (complete or charcoal stripped as required), then filter sterilized into appropriate MCF10a media (complete, or starvation media as indicated, see below) at a 1/50 final dilution, before adding to cells in culture for indicated times. Final supplement concentrations were 14.8 μM arachidonic acid and 17 μM BSA.

### 2.3 MCF10a culture

MCF10a (provided by Horizon Discovery (Cambridge, UK)) were maintained at 37 °C, 5% CO_2_, in DMEM/F12 media, supplemented with horse serum (5%, PAA), hydrocortisone (0.5 μg/ml), insulin (10 μg/ml), cholera toxin (0.1 μg/ml) and EGF (2 ng/ml). When required, cells were starved in DMEM/F12 media supplemented with charcoal-stripped horse serum (1%, PAA) hydrocortisone and cholera toxin. Early passages of cells were used (13–20).

For initial arachidonic acid loading time course, 1 (8 and 12 h) and 0.8 (24 h) x 10^5^ cells were plated to 3.5 cm tissue culture dishes in complete media in the absence or presence of arachidonic acid supplementation and incubated for the indicated times. For stimulation experiments, 1 × 10^5^ cells were plated onto 3.5 cm tissue culture dishes and allowed to adhere for 8 h in complete media. Cells were then incubated for a further 16 h in the absence or presence of arachidonic acid, followed by 6 h starvation (in the absence or presence of arachidonic acid) where indicated. Cells were stimulated with EGF (2 ng/ml) for 90 s at 37 °C. Media was rapidly aspirated and 1 M HCl (0.5 ml, 4 °C) added. Cells were scraped from the plates, collected and spun (15,000 g, 5 min, 4 °C) then supernatants removed. Pellets were then resuspended in H_2_O (170 μl) and quench mix (750 μl) and extraction commenced as described below.

### 2.4 Mass spectrometry measurements of inositol lipids

Mass spectrometry was used to measure inositol lipid levels essentially as previously described (Clark et al., 2011), using a QTRAP 4000 (AB Sciex) mass spectrometer and employing the lipid extraction and derivitization method described for MCF10a cells, with the modification that 10 ng C17:0/C16:0 PtdIns(3,4,5)P_3_ ISD was added to primary extracts and that final samples were dried in a speedvac concentrator rather than under N_2_. Measurements were conducted in duplicate per experiment.

## 3 Results

**Fig. 3:**
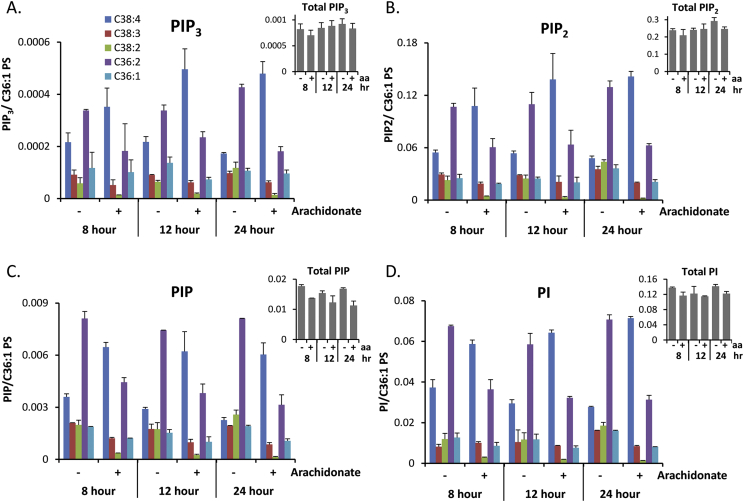
The effect of arachidonate supplementation on the molecular species of phosphoinositides in MCF10a cells. The five major species of PIP_3_ (A), PIP_2_ (B), PIP (C) and PI (D) present in MCF10a cells were quantified by HPLC-ESI mass spectrometry. Cells were incubated in complete medium with or without 14.7 μM arachidonate for the indicated times, as described in the methods. Data is shown for each phosphoinositide species corrected for amount of C36:1 phosphatidylserine in the same sample (mean ± SEM, n = 3). Insets show total combined species of phosphoinositides for each condition. aa = arachidonate.

Previous work in our laboratory has used HPLC-ESI mass spectrometry to detect the presence of multiple species of PI, PIP, PIP_2_ and PIP_3_ in MCF10a cells gown under standard cell culture conditions (Clark et al., 2011, Kielkowska et al., 2014); our current techniques do not allow us to differentiate between regio-isomers of the same mass e.g. PI(4)P vs PI(3)P vs PI(5)P. The distribution of molecular species was found to be very similar between the different classes of phosphoinositide, thus the most abundant species was C36:2, followed in decreasing order by C38:4, C36:1, C38:3 and C38:2 (Fig. 3).

We supplemented the culture medium with 17 μM BSA loaded with 14.8 μM arachidonate, to approximate the physiological levels of arachidonate that might be anticipated to prevail in the extracellular milieu *in vivo*. Supplementation with arachidonate increased the proportion of C38:4 species of phosphoinositide from around 25%–50% within 8 h, rising to 60% (after 24 h; Fig. 3). The relative increases in the C38:4 species were again very similar between phosphoinositide classes but, importantly, the total sum of all molecular species measured for PI, PIP, PIP_2_ and PIP_3_ remained remarkably constant with or without arachidonate supplementation (Fig. 3, insets).

**Fig. 4:**
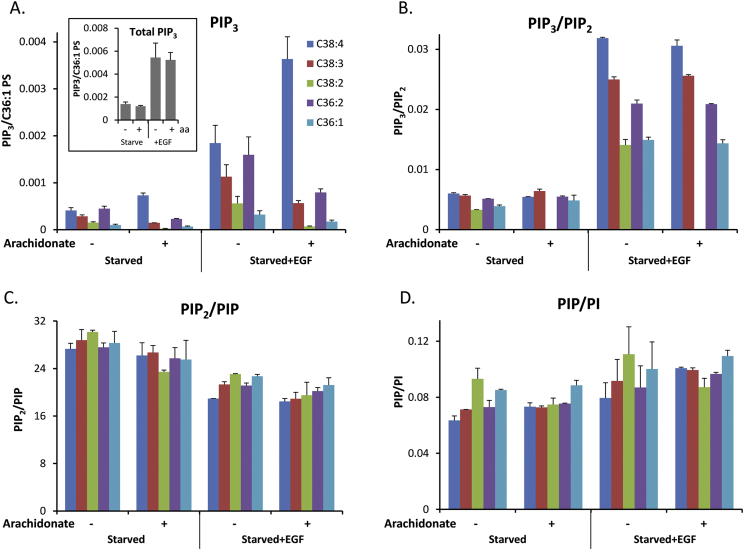
The effect of EGF on PIP_3_ accumulation in MCF10a cells grown in the presence or absence of arachidonate supplement. (A) Cells were incubated with or without 14.8 μM arachidonate for 24 h (including 6 h starvation) before stimulation with 2 ng/ml EGF or vehicle for 90 s. Data is shown for each PIP_3_ species corrected for amount of C36:1 phosphatidylserine in the same sample (mean ± SEM, n = 3). Inset displays total combined species of PIP_3_ under each condition. PIP_3_/PIP_2_ (B), PIP_2_/PI (C) and PIP/PI (D) ratios calculated from experiments described in panel A. Levels of PIP_3_ and PIP_2_ for C38:2 species under archidonate loading conditions were too low to obtain accurate PIP_3_/PIP_2_ calculations and were thus omitted.

We then investigated the impact of arachidonate supplementation on the amount and type of PIP_3_ species that accumulate after activation of Class I PI3K by EGF. Stimulation with 2 ng/ml EGF for 90 s, following starvation, stimulated a very similar rise in the total species of PIP_3_ which accumulate in the presence or absence of arachidonate supplementation (Fig. 4A). A careful analysis of the relative proportions of PIP_3_ molecular species which accumulated compared to the PIP_2_ species present in the same cells, suggests the following rank order of preference for accumulation of PIP_3_: C38:4 > C38:3 > C36:2 > C38:2 = C36:1 (Fig. 4B). No equivalent distortion was seen for the relative levels of PIP_2_ species compared to PIP species, or PIP species compared to PI species (Fig. 4C and D).

## 4 Discussion and conclusions

The substantial effect of arachidonate supplementation to increase the proportion of C38:4 molecular species of phosphoinositides in MCF10a cells supports the idea that cells grown in relatively small amounts of horse or calf serum are effectively arachidonate deprived (Rouzer et al., 2006). Arachidonate is obtained directly through the diet or indirectly via elongation and desaturation of the essential omega-6 fatty acid precursor, linoleic acid (Guillou et al., 2010). Most synthesis of arachidonate is thought to occur in the liver and is then distributed around the body complexed to albumin or esterified to lipids and proteins. Thus, many cells in culture are expected to possess limited capacity to synthesise arachidonate and this, combined with the degradation of linoleic and arachidonate through prolonged storage of serum, probably accounts for arachidonate deficiency in most mammalian tissue culture.

The mechanisms that drive relative enrichment of C18:0 at the sn-1 and C20:4 at the sn-2 positions of phosphoinositides are still poorly understood (Fig. 2B) (Anderson et al., 2013, D'Souza and Epand, 2014). There is evidence of significant C38:4 selectivity in the conversion of DG species to both PA and CDP-DG destined for PI synthesis but there appears to be little acyl selectivity in the subsequent conversions to PI (D'Souza and Epand, 2014, D'Souza and Epand, 2015). There is also good evidence that newly synthesised PA and PI can undergo cycles of deacylation and reacylation catalysed by PLA1s, PLA2s and acyl-CoA-transferases with substantial selectivity for their substrates e.g. LPIAT and LYCAT for incorporation of C20:4 and C18:0 into the sn-2 and sn-1 positions of PI, respectively (Anderson et al., 2013, Imae et al., 2012, Lee et al., 2012). Our data showing very similar total phosphoinositide pool sizes in the presence or absence of arachidonate supplementation suggest a combination of limited substrate selectivity and kinetic poise, coupled with successive cycles of synthesis/degradation, allows enzymes involved in the creation of different PI species to concentrate C38:4 species of phosphoinositides when appropriate fatty acids are present, but ensure similar total levels of the these important lipids are created when they are limiting. Further, the similar relative levels of different molecular species between steady-state pools of PI, PIP, PIP_2_ and PIP_3_, under both arachidonate depleted and supplemented conditions, suggest that the kinases and phosphatases involved in their inter-conversion do not exhibit profoundly different acyl selectivities with respect to the major species present (of note, C32:0 is a poor substrate for PI4P5Ks (Shulga et al., 2012)).

Stimulation of starved MCF10a cells with EGF induced a very similar accumulation of total PIP_3_ species in both arachidonate limited or supplemented conditions. However, we did observe a significant enrichment for some species of PIP_3_ compared to their corresponding levels of PIP_2_. This suggests that under conditions of acute Class I PI3K stimulation some differential substrate selectivity/availability is revealed with respect to acyl chain composition for enzymes synthesising or degrading PIP_3_ (ie Class I PI3Ks or phosphatases such as PTEN or SHIP1/2). It might be predicted that changes in nutrient availability could affect the acyl composition of PIP_3_ and other phosphoinositides. Further, a recent study has argued that p53 mutational status can determine phosphoinositide acyl composition independent of culture conditions (Naguib et al., 2015). Thus it will be important in future studies to understand how changes in the acyl composition of phosphoinositides might affect their ability to act as regulatory molecules.

## Conflicts of interest

The authors declare no conflicts of interest.
